# Alterations in Parafoveal and Optic Disc Vessel Densities in Patients with Obstructive Sleep Apnea Syndrome

**DOI:** 10.1155/2020/4034382

**Published:** 2020-01-10

**Authors:** Turgay Ucak, Ethem Unver

**Affiliations:** ^1^Department of Ophthalmology, Faculty of Medicine, Erzincan University, Erzincan, Turkey; ^2^Department of Pulmonology, Faculty of Medicine, Erzincan University, Erzincan, Turkey

## Abstract

**Purpose:**

To analyze the effects of obstructive sleep apnea syndrome (OSAS) on ocular parameters and determine the alterations in macular vasculature by optical coherence tomography-angiography (OCT-A) in patients with different stages of OSAS.

**Methods:**

All the participants underwent a full ophthalmological examination. Using the macular OCT-A scans, the retinal peripapillary capillary plexus (RPCP), foveal avascular zone (FAZ), and superficial and deep vessel densities were recorded.

**Results:**

A total of 77 patients (154 eyes) with OSAS and 27 control cases (54 eyes) were included in this prospective study. Of the OSAS patients, 27 had mild, 24 had moderate, and 26 had severe disease. The intraocular pressure (IOP) values were significantly higher in the severe OSAS group than the control cases (*p* = 0.001). The average retinal nerve fiber layer (RNFL) thickness and the RNFL thickness of the temporal and inferior quadrants were significantly lower in the severe OSAS group compared with the control cases (*p* = 0.001). The average retinal nerve fiber layer (RNFL) thickness and the RNFL thickness of the temporal and inferior quadrants were significantly lower in the severe OSAS group compared with the control cases (*p* = 0.001). The average retinal nerve fiber layer (RNFL) thickness and the RNFL thickness of the temporal and inferior quadrants were significantly lower in the severe OSAS group compared with the control cases (

**Conclusions:**

Decreased vascular structures and increased FAZ may also be associated with the disease severity in OSAS and may be the main pathophysiological mechanisms in ocular alterations, which should be investigated in further studies.

## 1. Introduction

Obstructive sleep apnea syndrome (OSAS) is characterized by the recurrent narrowing of the upper airway, causing intermittent oxyhemoglobin desaturation, sleep fragmentation, and daytime sleepiness [1]. OSAS is a major public health burden because of its high prevalence, affecting almost 6–13% of the population [2, 3]. It has been shown that chronic intermittent hypoxia in OSAS patients is associated with systemic inflammation leading to the systemic effects of OSAS [4, 5].

OSAS is a systemic disease disturbing many organs and tissues. Although there are some studies reporting the association of OSAS with glaucoma or decreased retinal nerve fiber layer (RNFL) thickness, the data regarding the ocular alterations in OSAS are limited and conflicting [6–8].

With an increase in OSAS prevalence, its systemic complications are also gaining importance. Intermittent hypoxia in OSAS was associated with oxidative stress, sympathetic hyperactivation, disturbed vasomotor reactivity, and vascular inflammation [9]. In this study, we aimed to analyze the effects of OSAS on ocular parameters and determine the alterations in ocular vasculature in patients with different stages of OSAS. By this way, we aimed to define the predictive measures if required in OSAS patients associated with ocular alterations.

## 2. Materials and Methods

The study was approved by the Ethics Committee of Erzincan University and performed in accordance with the principles of the Helsinki Declaration. Informed consent was obtained from all participants.

Consecutive patients that underwent polysomnography at the Sleep Disorders Center of Erzincan University Training and Research Hospital between January and August 2018 were enrolled in this study. A 3% decline in SpO_2_ and/or an arousal associated with a ≥30% reduction in airflow lasting at least 10 seconds was defined as hypopnea. Apnea/hypopnea index (AHI) was identified, and the patients with OSAS were categorized into mild (AHI ≥5 and <15), moderate (AHI ≥15 and <30), and severe (AHI ≥30) OSAS groups [10]. In the control group, all patients had an AHI of less than 5.

All the participants underwent a full ophthalmological examination, including auto-refraction (Tonoref III, Nidec Co. Ltd, Aichi, Japan), best-corrected visual acuity (BCVA), intraocular pressure (IOP) measurement by Goldmann applanation tonometry (mmHg), slit lamp biomicroscopy, macular and peripapillary thickness measurement by a spectral domain-optical coherence tomography (SD-OCT) (RS-3000 Advance, Nidek Co. Ltd, Aichi, Japan), and quantification of the foveal avascular zone (FAZ) and vessel density (VD) by optical coherence tomography-angiography (OCT-A) (RS-3000 Advance AngioScan, Nidek Co. Ltd, Aichi, Japan).

### 2.1. Scan Protocol

We used Nidec's RS-3000 Advance and Navis Ex. Ver. 1.1.5 software to collect and analyze the SD-OCT and OCT-A images. The device operates at a central wavelength of 880 nm and a speed of A-scan rate of 53,000 second/s. The axial and transverse scan resolution in tissue is 7 and 20 *μ*m, respectively. The scan range of the device ranges from 3 to 12 mm horizontally, 3 to 9 mm vertically, and 2.1 mm in depth.

**Figure 1:**
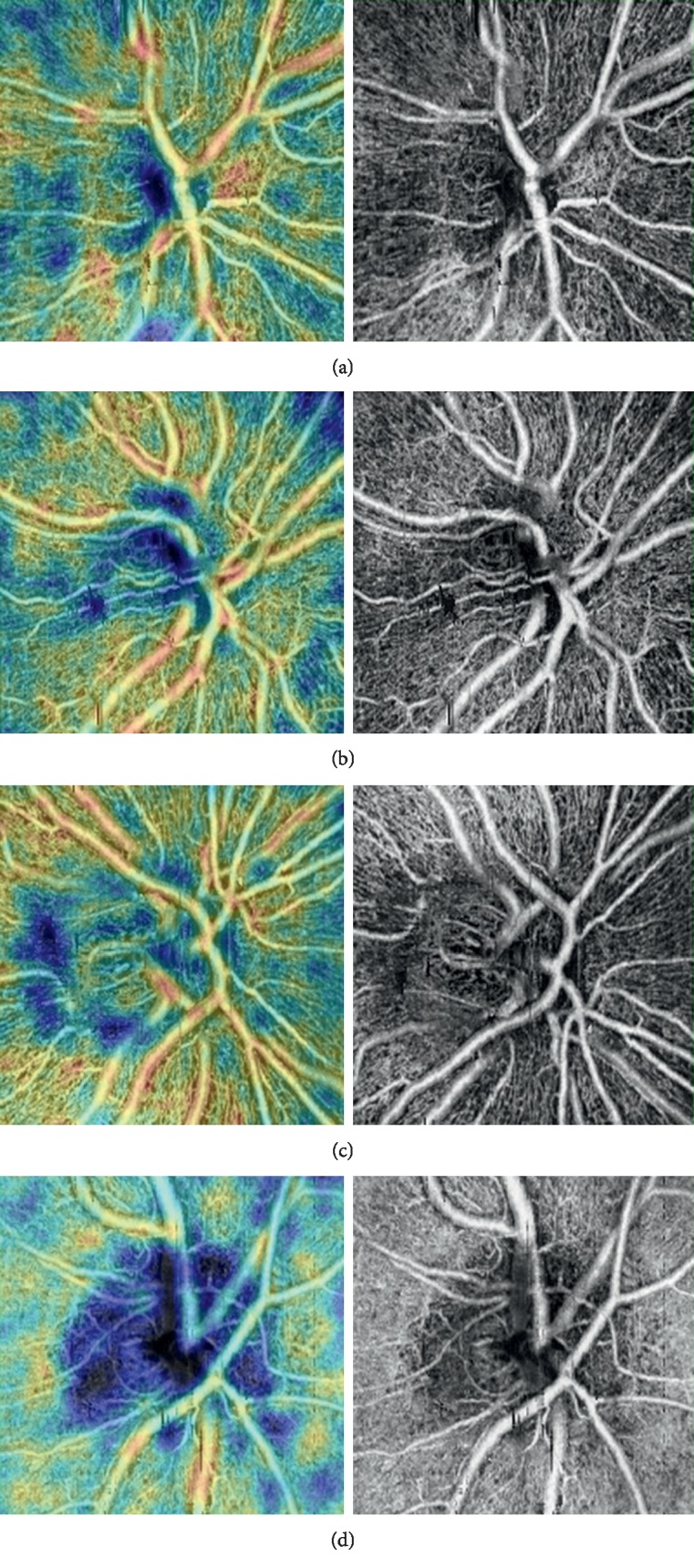
Peripapillary coherence tomography-angiography images: (a) control; (b) mild OSAS; (c) moderate OSAS; and (d) severe OSAS.

**Figure 2:**
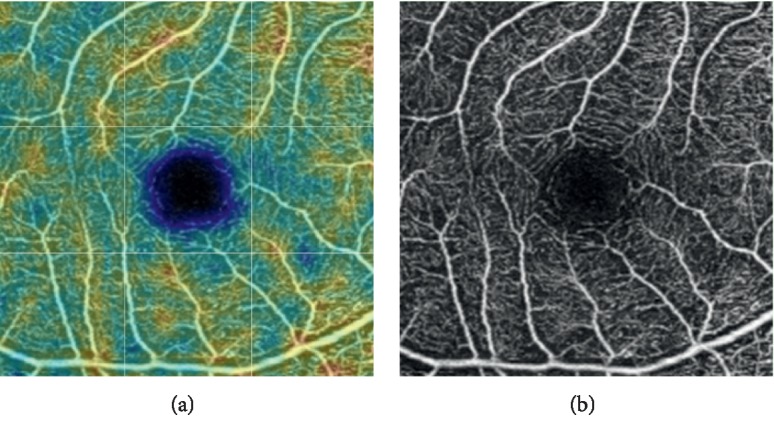
Example of superficial macular capillary density measurements (control group, right eye).

**Figure 3:**
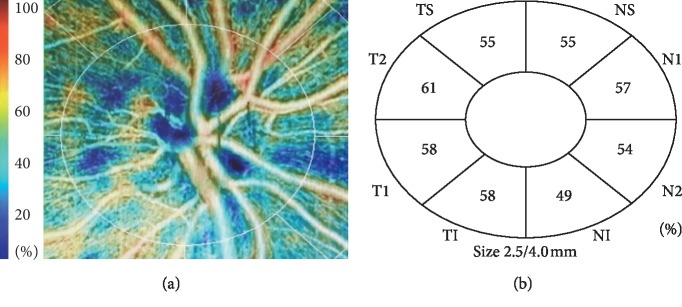
Example of peripapillary density measurements. NS: nasal superior; N: nasal; NI: nasal inferior; TI: temporal inferior; T: temporal; TS: temporal superior.

Each eye was dilated with 1% tropicamide eye drop (Tropamid, Bilim Ilac Ltd., Istanbul, Turkey) and scanned by an experimented operator. The scanning was repeated if the SSI quality of the image was <7/10. The scans included a 3 × 3 mm^2^ macula map centered on the fovea and a 2.4 × 4 mm^2^ disc map centered on the optic nerve head. The following data were recorded after the scanning was completed: macular thickness, ganglion cell complex (GCC) thickness, RNFL thickness, FAZ area, and the VDs of the macular and peripapillary areas (Figures 1–3). The RNFL thickness was recorded at four different quadrants using the temporal-superior-nasal-inferior-temporal (TSNIT) map. Nine regions of the Early Treatment Diabetic Retinopathy Study (ETDRS) map was used to obtain the macular thickness. The GCC thickness was recorded for the whole and superior/inferior sectors of the macula. The VD of the retinal peripapillary capillary plexus (RPCP) was recorded for the S/I, and TSNIT sectors were recorded using the OCT-A scans of the peripapillary area.

### 2.2. Statistical Analyses

These analyses were performed using SPSS software version 20.0 (SPSS, Inc., Chicago, IL, USA). The continuous variables were presented as mean ± standard deviation values. One-way analysis of variance was used to compare the age between the groups. The Pearson chi-squared test used to analyze the gender distribution among the groups. The correlations between AHI and the mean VD value of left and right eyes were analyzed using the Pearson correlation coefficient. A single GEE model, corrected for age and gender, with working correlation matrix “independent” was created for each parameter as a dependent variable and the study groups as the main effect (ordinal factor) to account for within-individual intereye effects. GEE results are given with regression coefficient (B) and 95% Wald confidence interval (CI). *P* < 0.05 was considered statistically significant.

## 3. Results

A total of 77 patients (154 eyes) with OSAS and 27 control cases (54 eyes) were included in this prospective study. Of the OSAS patients, 27 had mild, 24 had moderate, and 26 had severe disease. The demographic features of the study participants are summarized in Table 1. There was no significant difference between the study groups regarding the BCVA or central macular thickness (CMT) values (Table 2). However, the IOP value was significantly higher in the severe OSAS group than in the control cases (*p* = 0.001) (Table 2).

The average RNFL thickness and the RNFL thicknesses of the temporal and inferior quadrants were significantly thinner in the severe OSAS group (Table 2). Both superior and inferior GCC thicknesses were also determined to be significantly lower in the moderate and severe OSAS groups compared with the control cases.

There was a decrease in the macular thickness (ETDRS) with the presence and increased severity of OSAS. When compared with the control group, this decrease was statistically significant in the severe OSAS group in outer superior, outer nasal, and outer temporal measurements (Table 3). When compared with the control group, this decrease was statistically significant in both moderate and severe OSAS groups in outer inferior measurement.

The radial papillary capillary network was analyzed and compared between the control and OSAS groups. There was a significant decrease in the mean, nasal 2, and temporal 2 RPCP values of the moderate and severe OSAS patients compared with the control group (Table 4). There was a significant decrease in the nasal 1 and temporal 1 RPCP values of the severe OSAS patients compared with the control group (Table 4).

There was a significant increase in the FAZ values in the severe OSAS patients compared with the control group (*p*=0.001). Superficial VD was significantly lower in the severe OSAS group compared with the control cases (*p*=0.006). Deep VD was significantly lower in all OSAS groups compared with the control cases (Table 5).

Table 6 presents the results of correlation analysis performed in the OSAS groups between AHI and the other investigated parameters, namely, IOP, RNFL thickness, superior and inferior GCC thicknesses, RPCP, FAZ, and superficial and deep VDs. There was a weak positive correlation between AHI and FAZ (*r* = 0.257, *p*=0.039) and weak negative correlations between AHI and superficial and deep VDs (*r* = −0.245, *p*=0.045 and *r* = −0.236, *p*=0.048, respectively).

## 4. Discussion

In this study, we analyzed the ocular alterations in patients with different stages of OSAS and determined that OCTA-FAZ increased and superficial and deep VDs and RPCP values decreased in the OSAS patients and these alterations were more severe with the increasing disease stage and were correlated with AHI. We did not determine any effects of OSAS on BCVA or CMT, but IOP was higher in severe OSAS patients.

Systemic hypoxia is known to affect retinal perfusion and retinal ganglion cells [11]. Zhang et al. [12] reported that in moderate and severe OSAS groups, the inner diameter of the ophthalmic artery was significantly decreased and that of the posterior ciliary artery was significantly increased. The authors reported that the blood flow of the ophthalmic posterior ciliary and central retinal arteries changed with the disturbed autoregulation of the latter two arteries in OSAS cases. Yu et al. [13] investigated the alterations in the retinal vasculature in patients with OSAS and reported that in the advanced form of OSAS, the RNFL thickness did not differ, but the VDs of the parafoveal and peripapillary areas were negatively correlated with AHI due to the significant reduction in the VDs of this group. Karakucuk et al. [14] analyzed the resistivity index in the ophthalmic and central retinal arteries and determined that there was no statistically significant difference between the OSAS patients and control cases. Moyal et al. [15] reported that the VDs of the macula did not significantly differ between the OSAS patients and control cases. The authors included OSAS cases from all stages (mild, moderate, and severe). They also did not determine any alterations in the RNFL or GCC thicknesses in the OSAS patients compared with the control cases.

We determined a significant increase in the FAZ values in the severe OSAS patients with a significant decrease in superficial VD while deep VD was significantly lower in all OSAS groups compared with the control cases.

To the best of our knowledge, this is the first study evaluating the RPCP values in OSAS patients. RPCP was determined to be decreased in OSAS, being more prominent in advanced stages of the disease. This decrease in the capillary plexus may be associated with the decreased vascular growth factors and may play a role in the pathophysiological alterations of ocular structures in OSAS [16]. Supporting this data, we also determined a significant increase in FAZ and significant decrease in both superficial and deep VDs in severe OSAS patients. Moreover, FAZ was positively correlated and VDs were negatively correlated with AHI. There is limited data in the literature concerning the alterations in FAZ or VDs in OSAS. In a very recent study, Ye et al. [17] reported a significant decrease in the FAZ of the superficial capillary plexus but a significant increase in the deep capillary plexus after adenoidectomy in children. Regarding these results, we can conclude that quantitative OCT-A parameters reveal macular ischemia at both superficial and deep retinal capillary plexuses, which may play an important role in the pathophysiology of ocular alterations in severe OSAS patients. Prospective studies are warranted to investigate the alterations in posttreatment OCT-A parameters in OSAS cases to elucidate the exact role of these alterations in clinical practice.

In chronic hypoxic conditions, deficiencies in nutrients or oxygen and more importantly increased production of reactive oxygen species may cause severe problems in ocular tissues, which are highly susceptible to oxidative stress [18, 19]. In the literature, there are many studies about the effects of OSAS on ocular structures; however, the results are conflicting. We did not determine any significant changes in BCVA or CMT, but IOP significantly increased in patients with severe OSAS. The literature contains conflicting data regarding the alterations in IOP in OSAS cases. For example, Karakucuk et al. reported an increase in glaucoma rates in OSAS and a positive correlation between the AHI and IOP values [14], whereas Moghimi et al. [20] found higher IOP, worse visual field indices, and lower RNFL parameters in OSAS cases compared with the control group and revealed a significant positive correlation between AHI and IOP. In another study, Cohen et al. [21] observed a significant rise in IOP during the sleep period in OSAS patients, which was not affected by continuous positive airway pressure (CPAP) therapy. Shinmei et al. [22] evaluated the IOP changes during nocturnal sleep in patients with OSAS and reported that the mean IOP levels during apnea events were lower than during nonapnea phases. Nadarajah et al. [23] did not determine any significant alteration in the IOP values in patients with OSAS compared with the control cases, but they defined decreased corneal hysteresis in OSAS, which may be another link between this disease and the development of glaucoma. Teberik et al. [24] reported that there were no significant differences between the OSAS patients and control cases regarding the IOP, central corneal thickness, or RNFL values in all quadrants. These conflicting results in the literature may be associated with the varying severity of OSAS and disease duration of the samples. In the current study, we determined an increase in the IOP values in OSAS patients, and this increase was statistically significant in the severe disease group.

There was a decrease in the RNFL thickness in the OSAS patients, and this decrease was even more prominent in the advanced stages of the disease. Similarly, in a meta-analysis of six studies evaluating a total of 1,034 eyes, it was reported that OSAS resulted in a decrease in the RNFL thickness and the greater the severity of OSAS, the greater the loss of RNFL. Moreover, the most and least affected quadrants were the inferior and temporal quadrants, respectively [25]. In contrast, in our study, both the inferior and temporal quadrants were found to be most affected. However, there were some recent studies that did not determine any thinning in RNFL in OSAS. For instance, Adam et al. [26] did not find any significant alteration in the RNFL thickness in OSAS patients. Similarly, Yuvaci et al. [27] reported no significant difference between the OSAS patients and control cases regarding CMT or RNFL thicknesses. The authors also noted that with the treatment of severe OSAS patients, statistically significant thinning of RNFL was detected at the end of the 12th week. Kücük et al. [28] evaluated the right eyes of 45 OSAS cases and 43 age- and sex-matched healthy controls and reported that there were no significant differences between the two groups regarding IOP, RNFL thickness, subfoveal and parafoveal choroidal thicknesses, and peripapillary choroidal thickness. Research suggests that RNFL is thinner in patients with OSAS although this does not reach a statistically significant level in all studies. These varying significance levels may be due to the differences in the number of patients and severity of the disease. In this study, we also found a significant decrease in the RNFL thickness, and the thinnest RNFL was obtained from the severe OSAS group.

There are some limitations of this study that should be mentioned. Firstly, we did not analyze some clinical parameters, e.g., body mass index or laboratory tests, such as insulin resistance and fasting blood glucose levels, which may also cause some alterations in the ocular structures of OSAS patients. Secondly, we did not record the disease duration in our sample.

In conclusion, OSAS has diverse effects on ocular structures, which should be kept in mind in clinical practice, since the prevalence of this disease is increasing. Elevated IOP levels and decreased RNFL thickness may increase the glaucoma incidence in this patient group; thus, these cases should be monitored regularly. Decreased vascular densities and increased FAZ may also be associated with the severity of OSAS and may be the main pathophysiological mechanisms in ocular alterations, which should be investigated in further studies.

## Figures and Tables

**Table 1:** Comparison of the demographic features between the OSAS and control groups.

	Control (*n* = 27, 54 eyes)	Mild OSAS (*n* = 27, 54 eyes)	Moderate OSAS (*n* = 24, 48 eyes)	Severe OSAS (*n* = 26, 52 eyes)	*p*
Age	52.89 ± 11.60	52.17 ± 11.36	55.91 ± 8.96	55.42 ± 9.44	0.899
Gender (M/F)	14/13	15/12	14/10	15/11	0.977

M: male, F: female, OSAS: obstructive sleep apnea syndrome.

**Table 2:** Comparison of the age and sex corrected IOP, CMT, RNFL, and GCC thicknesses of OSAS groups with healthy controls using generalized estimating equation models.

	Control (*n* = 27, 54 eyes)	Mild OSAS (*n* = 27, 54 eyes)	Moderate OSAS (*n* = 24, 48 eyes)	Severe OSAS (*n* = 26, 52 eyes)
BCVA (Snellen)	0.94 ± 0.085	0.96 ± 0.08 −0.003 −0.049/0.042 (0.885)	0.94 ± 0.08 0.015 −0.028/0.059 (0.490)	0.94 ± 0.09 0.004 −0.043/0.058 (0.848)

IOP (mmHg)	12.13 ± 1.02	13.11 ± 2.25 0.072 −0.431/0.535 (0.779)	13.58 ± 1.85 0.827 −0.695/1.340 (0.065)	13.91 ± 1.96 1.151 0.765/1.73 (**0.001)**

CMT	261.57 ± 41.40	267.01 ± 24.26 5.444 −9.979/20.869 (0.489)	265.70 ± 22.12 4.128 −10.990/19.246 (0.593)	259.50 ± 24.43 −2.074 −17.752/13.374 (0.792)

Average RNFL (*μ*)	109.52 ± 11.57	103.73 ± 8.98 −2.788 −8.030/2.455 (0.297)	100.23 ± 10.55 −5.284 −11.484/−0.920 (0.095)	99.26 ± 12.06 −6.249 −12.398/−0.100 (**0.046)**

Superior RNFL (*μ*)	129.12 ± 18.17	128.30 ± 16.15 0.178 −7.899/8.255 (0.966)	122.10 ± 19.61 −7.023 −16.237/2.191 (0.135)	124.19 ± 19.75 −4.937 −14.131/4.256 (0.293)

Nasal RNFL (*μ*)	79.34 ± 18.32	77.97 ± 15.98 2.439 −5.377/10.254 (0.541)	76.90 ± 17.30 1.071 −6.517/8.659 (0.782)	71.69 ± 15.24 −5.215 −12.646/2.216 (0.169)

Inferior RNFL (*μ*)	137.92 ± 20.06	132.98 ± 14.87 −4.945 −13.287/3.393 (0.245)	126.0 ± 19.17 −9.464 −19.322/0.393 (0.060)	124.46 ± 21.53 −11.926 −21.329/−2.216 (**0.013)**

Temporal RNFL (*μ*)	78.90 ± 15.14	69.11 ± 9.93 −4.290 −11.991/3.410 (0.245)	64.61 ± 11.85 −6.580 −13.213/0.520 (0.052)	62.32 ± 10.94 −9.792 −15.529/−4.055 (**0.001)**

GCC superior (*μ*)	111.40 ± 9.32	107.40 ± 9.33 −2.004 −7.272/3.265 (0.456)	103.89 ± 6.75 −5.514 −10.324/0.703 (**0.025)**	103.70 ± 9.37 −5.504 −11.028/3.813 **(0.041)**

GCC inferior (*μ*)	110.98 ± 11.64	106.57 ± 10.64 −2.405 −8.076/3.267 (0.406)	103.44 ± 8.96 −5.535 −10.806/−0.214 (**0.040)**	101.75 ± 17.45 −7.231 −13.991/−0.472 (**0.031)**

OSAS: obstructive sleep apnea syndrome; BCVA: best-corrected visual acuity; IOP: intraocular pressure; CMT: central macular thickness; RNFL: retinal nerve fiber layer; GCC: ganglion cell complex. *p* values of comparisons between the control group are represented within parentheses.

**Table 3:** Comparison of the age- and sex-corrected macular thickness (ETDRS) values of OSAS groups with healthy controls using generalized estimating equation models.

Macula thickness outer ring (6 mm) and inner ring (3 mm)	Control (*n* = 27, 54 eyes)	Mild OSAS (*n* = 27, 54 eyes)	Moderate OSAS (*n* = 24, 48 eyes)	Severe OSAS (*n* = 26, 52 eyes)
Outer ring superior	329.81 ± 19.86	326.30 ± 17.66 −3.507, −12.823/5.809 0.461	323.84 ± 23.87 −5.969, −17.091/5.154 0.293	319.38 ± 15.94 −10.432, −19.632/−1.232 **0.026**

Outer ring nasal	341.68 ± 20.91	333.96 ± 18.21 −7.724, −17.543/2.095 0.123	331.61 ± 14.83 −8.204, −19.219/2.810 0.144	330.48 ± 12.30 −8.504, −19.314/−0.823 **0.033**

Outer ring inferior	330.94 ± 20.81	322.11 ± 25.12 −8.820, −20.302/2.643 0.131	317.87 ± 22.57 −13.072, 23.989/2.158 **0.019**	316.09 ± 19.49 −12.848, −24.555/-1.142 **0.031**

Outer ring temporal	323.74 ± 20.70	317.76 ± 21.28 −5.972, −16.404/4.464 0.262	308.70 ± 19.70 −8.625, −19.516/2.265 0.121	305.11 ± 15.09 −15.039, −25.358/−4.720 **0.004**

Inner ring superior	347.24 ± 21.75	342.69 ± 16.33 −4.548, −14.394/5.297 0.365	340.63 ± 12.34 −6.602, −15.527/2.322 0.147	336.53 ± 12.76 −10.702, −22.111/0.706 0.066

Inner ring nasal	345.27 ± 20.84	334.48 ± 48.04 −10.797, 26.693/5.099 0.183	338.80 ± 14.70 −6.469, −15.267/2.328 0.150	336.15 ± 20.99 −9.124, −19.982/1.734 0.100

Inner ring inferior	343.31 ± 22.81	338.40 ± 20.32 −4.911, −15.826/2.328 0.378	333.91 ± 19.68 −9.400, −19.878/1.078 0.079	331.86 ± 23.84 −11.449, −23.209/0.310 0.056

Inner ring temporal	330.40 ± 19.50	327.94 ± 18.01 −2.465, −11.947/7.017 0.610	325.78 ± 14.50 −4.620, −13.052/3.812 0.283	322.34 ± 20.08 −8.061, −18.075/1.953 0.115

Macula map regions within a 6 × 6 mm area centered on the fovea, as defined by the ETDRS. *p* values of comparisons between the control group are represented within parentheses.

**Table 4:** Comparison of the age- and sex-corrected RPCP values of OSAS groups with healthy controls using generalized estimating equation models.

	Control (*n* = 27, 54 eyes)	Mild OSAS (*n* = 27, 54 eyes)	Moderate OSAS (*n* = 24, 48 eyes)	Severe OSAS (*n* = 26, 52 eyes)
RPCP	55.65 ± 3.33	53.73 ± 3.80 −0.923 −2.471/0.625 (0.243)	51.67 ± 4.01 −2.837 −4.508/−1.166 (**0.001)**	51.09 ± 3.37 −2.563 −3.919/−1.208 **(0.001)**
Nasal superior	54.09 ± 4.98	52.67 ± 7.14 −0.561 −2.743/1.622 (0.824)	51.32 ± 6.95 −2.633 −5.473/0.206 (0.069)	52.87 ± 5.08 −1.420 −4.187/1.335 (0.311)
Nasal 1	54.74 ± 5.91	52.69 ± 8.64 −2.054 −5.456/1.347 (0.236)	52.94 ± 7.01 −1.660 −4.722/1.403 (0.288)	50.48 ± 5.69 −3.741 −6.209/−1.273 (**0.003)**
Nasal 2	51.20 ± 4.78	50.14 ± 6.34 −1.066 −3.497/1.364 (0.390)	47.00 ± 9.66 −4.042 −7.845/−0.238 (**0.037)**	47.34 ± 5.61 −3.225 −5.399/−1.051 (**0.004)**
Nasal inferior	56.09 ± 5.23	55.31 ± 6.14 −0.779 −3.497/1.508 (0.504)	54.37 ± 6.25 −3.444 −6.398/−0.490 (0.122)	54.40 ± 4.98 −1.050 −2.832/0.732 (0.248)
Temporal inferior	57.69 ± 6.36	56.63 ± 7.11 −1.058 −3.735/1.620 (0.439)	56.10 ± 6.39 −1.415 −4.374/1.544 (0.348)	54.87 ± 5.78 −2.217 −4.744/0.309 (0.085)
Temporal 1	51.61 ± 4.91	50.00 ± 9.13 −1.611 −5.008/1.785 (0.353)	49.51 ± 6.51 −2.017 −4.471/0.438 (0.107)	46.82 ± 7.66 −4.271 −6.952/−1.599 (**0.002)**
Temporal 2	55.96 ± 4.28	53.86 ± 7.87 −2.100 −4.925/0.725 (0.145)	50.97 ± 8.52 −4.855 −7.499/−2.211 **(0.001)**	52.82 ± 5.30 −2.495 −4.243/0.747 (**0.005)**
Temporal superior	56.17 ± 4.60	56.06 ± 5.51 −0.107 −2.111/1.898 (0.717)	53.51 ± 7.29 −2.248 −5.201/0.705 (0.136)	55.17 ± 6.64 −0.507 −2.488/1.474 (0.616)

RPCP: radial peripapillary capillary network. *p* values of comparisons between the control group are represented within parentheses.

**Table 5:** Comparison of the age- and sex-corrected FAZ and superficial and deep VDs of OSAS groups with healthy controls using generalized estimating equation models.

	Control (n = 27, 54 eyes)	Mild OSAS (*n* = 27, 54 eyes)	Moderate OSAS (*n* = 24, 48 eyes)	Severe OSAS (*n* = 26, 52 eyes)
OCT-A-FAZ	0.29 ± 0.12	0.32 ± 0.09 0.028 −0.024/0.081 (0.292)	0.38 ± 0.16 0.0.85 −0.004/0.174 (0.061)	0.40 ± 0.09 0.101 0.042/0.160 (**0.001)**
Superficial VD	44.13 ± 2.95	41.12 ± 3.15 −1.012 −2.412/0.388 (0.157)	40.32 ± 4.11 −1.807 −3.873/0.259 (0.086)	39.95 ± 3.52 −2.170 −3.729/−0.611 (**0.006)**
Deep VD	37.33 ± 4.38	33.49 ± 5.02 −3.843 −6.121/−1.566 (**0.001)**	31.42 ± 6.48 −5.905 −8.736/−3.074 (**0.001)**	30.19 ± 5.63 −7.140 −10.185/−4.095 (**0.001)**

FAZ: foveal avascular zone; VD: vessel density. *p* values of comparisons between control group are represented within parentheses.

**Table 6:** Results of the correlation analysis performed in the OSAS group between AHI and the other parameters.

	*R*	*p*
IOP	0.056	0.63
RNFL	0.015	0.89
GCC superior	0.131	0.25
GCC inferior	0.036	0.75
RPCP	0.067	0.58
FAZ	**0.257**	**0.039**
Superficial VD	**−0.245**	**0.045**
Deep VD	**−0.236**	**0.048**

IOP: intraocular pressure; RNFL: retinal nerve fiber layer; GC: ganglion cell complex; RPCP: radial peripapillary capillary network; FAZ: foveal avascular zone; VD: vessel density.

## Data Availability

The data supporting the results of the current article are available from the corresponding author upon request.
